# Understanding Chloride Diffusion Coefficient in Cementitious Materials

**DOI:** 10.3390/ma16093464

**Published:** 2023-04-29

**Authors:** Zhiyuan Xu, Guang Ye

**Affiliations:** Section of Materials and Environment (Microlab), Faculty of Civil Engineering and Geosciences, Delft University of Technology, 2628 CD Delft, The Netherlands; g.ye@tudelft.nl

**Keywords:** cementitious materials, diffusion coefficient, chloride transport, natural diffusion test

## Abstract

One of the key problems that affect the durability of reinforced concrete structures is the corrosion of rebar induced by chloride. Despite the complicated transport mechanism of chloride ions in cementitious materials, diffusion is still the key mechanism of chloride ingress. The determination of the chloride diffusion coefficient will help to predict the chloride profile inside the cementitious materials and estimate the service life with regard to chloride-induced corrosion. However, this paper shows that the chloride diffusion coefficient in the literature is sometimes misunderstood. Such a misunderstanding results in the overestimation of the chloride resistance of cementitious materials. To clarify the chloride diffusion coefficient, this paper first presents the steady- and non-steady-state diffusion equations in cementitious materials. The factors that influence the diffusive flux are identified. The effective and apparent diffusion coefficients are then clearly explained and properly defined. We also point out the obscure definitions of the effective diffusion coefficient in the literature. The varied definitions of the effective diffusion coefficient are the result of the consideration of different factors affecting the diffusion process. Subsequently, this paper discusses two natural diffusion test methods that are frequently employed in cementitious materials to measure the chloride diffusion coefficient. The influencing factors considered by the measured diffusion coefficients are analyzed in detail. Then, the diffusion coefficients determined in some of the studies are reviewed. It is shown that three typical errors could occur when numerically determining the diffusion coefficients.

## 1. Introduction

Cementitious materials, including cement-based materials and alkali-activated materials (AAMs) are, in essence, porous materials; thus, the ionic concentration difference between the pore solution and the external environment causes ion diffusion. Due to the porous nature of cementitious materials, the rate of transport of aggressive ions is a major factor influencing the durability of cementitious materials. Chloride-induced rebar corrosion is one of the critical durability issues for reinforced concrete structures [1]. In the absence of chloride ions, a protective passive layer is formed on the surface of the embedded rebar. This passive layer consists of Fe_3_O_4_ or Fe_2_O_3_ and is stable with oxygen and in a high alkaline environment [2]. In the presence of this passive layer, the rebar is protected against corrosion. However, the passive layer on the rebar surface can be broken down when the chloride concentration on the rebar surface reaches the threshold [2]. Thus, the rebar corrosion is initiated. This circumstance often occurs in reinforced concretes exposed to marine environments or de-icing salts, where chloride penetrates into the concrete cover.

Chloride ion transport in cementitious materials is a complex process. It involves multiple mechanisms, including diffusion, capillary suction, and pressure-induced flow [3]. In most practical situations, e.g., the marine environment, diffusion is the dominant mechanism for chloride ingress [4]. Thus, the chloride diffusion coefficient is applied to describe chloride resistance for cementitious materials. It can be interpreted as the flux per unit of concentration gradient due to diffusion. 

The diffusion coefficient is normally determined experimentally. Many experimental methods have been developed for this purpose. These methods can be categorized into two groups: natural diffusion tests [5,6,7,8] and migration tests [9,10]. The immersion test [5,6] and diffusion cell test [7,8] are two major methods used for natural diffusion tests. Compared to migration tests, natural diffusion tests are closer to real applications in the field, but they are always very time-consuming. Migration tests comprise the steady-state migration test [10], non-steady-state migration test [9], and electrical conductivity test [11,12]. Migration tests are known for the fast determination of the diffusion coefficient. However, because of the complex transport mechanisms for chloride ions in the electrical field, there are issues related to the interpretation of the diffusion coefficient determined by migration tests. It has been reported that the chloride diffusion coefficient measured through the non-steady-state migration test is ten times higher than that measured by the steady-state migration test [13].

Numerical methods have also played an important role in predicting the diffusion coefficient in cementitious materials [14,15,16,17]. In addition to the conventional physics-based numerical methods, machine learning techniques have also been employed in recent years to predict the chloride diffusion coefficients. Liu et al. utilized the artificial neural network technique to predict the chloride diffusion coefficient on the basis of a database consisting of 653 diffusion coefficient results [18]. Tran evaluated eight machine learning models in the prediction of the chloride diffusion coefficient of concrete with supplementary cementitious materials [19]. However, some inconspicuous errors can easily be introduced into these numerical methods and can significantly impair the predicted diffusion coefficient. These errors mainly result from the misunderstanding of the experimentally determined diffusion coefficient and the vague definition of several concepts, such as the effective diffusion coefficient and the chloride concentration. This paper intends to clarify these vague concepts that are related to the diffusion coefficient and to explain the diffusion coefficient measured in different natural diffusion test methods. Meanwhile, a few typical errors related to the numerically determined diffusion coefficient are analyzed.

## 2. Understanding Diffusion Coefficient

### 2.1. Diffusive Flux

**Figure 1:**
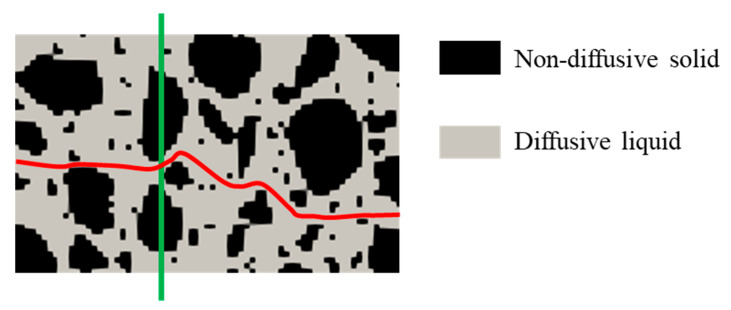
Schematic illustration of the effects of tortuosity and reduced cross-section area on diffusive flux. The black area and grey area refer to the non-diffusive and diffusive parts in cementitious materials. The red line represents a tortuous diffusive path. The green line represents a cross-section with a reduced pore solution area.

Chemical species in solution move as a result of their concentration gradient. This process is called diffusion. The famous Fick’s first law describes the rate of mass transfer (i.e., diffusive flux) with the concentration gradient, which can be expressed by:

(1) $$J=-D_{0}\frac{\partial C}{\partial x},$$

where *C* (mol L^−1^) represents the solute concentration in the pore solution; *x* (m) is the diffusion direction; *D*_0_ (m^2^ s^−1^) represents the solute diffusion coefficient; and *J* (m mol L^−1^ s^−1^) is the diffusive flux. Fick’s first law is only applicable to the solute transport in solution. In cementitious materials, chloride ions diffuse at a much slower rate than those in solution. This is because, on the one hand, the pathways for the chloride movement are more tortuous and, on the other hand, chloride ions can travel only in the pore solution, which occupies a mere part of the cross-section area [20]. Figure 1 displays these two effects. Under the assumptions of homogenous cementitious materials and one-dimensional diffusion, the chloride diffusive flux in cementitious materials, defined according to the total cross-section area, is given by [20].

(2) $$J=-D_{0}\tau \theta \frac{\partial C}{\partial x},$$

where θ (m^3^ of pore solution per m^3^ cementitious material) is the volumetric content of the pore solution in the cementitious materials, and τ is the tortuosity factor of the cementitious materials. The volumetric water content θ is defined by $\theta =\phi S_{w}$, where ϕ (m^3^ of pore per m^3^ cementitious material) is the porosity and $S_{w}$ is the water saturation degree. In saturated cementitious materials, θ is simply the porosity ϕ. It should be noted that porosity ϕ specifically refers to open porosity where the pores are interconnected and open to water. The tortuosity factor τ is used to characterize the degree of tortuous diffusive pathways. It can be expressed as [21]

(3) $$\tau ={\left(\frac{L_{s}}{\langle L_{d}\rangle }\right)}^{2},$$

where $L_{s}$ is the straight-line distance and $\langle L_{d}\rangle$ is the average length of the diffusive pathways.

As described in the Stokes–Einstein equation, the solute diffusion coefficient is highly dependent on solution viscosity. In cementitious materials, the pore solution viscosity is increased because of the adsorption of water molecules at the charged solid surfaces [22]. This then results in a smaller chloride diffusion coefficient in the pore solution. To incorporate the effect of increased pore solution viscosity on the chloride diffusion, Equation (2) is modified as follows:

(4) $$J=-{D^{\prime }}_{0}\tau \theta \frac{\partial C}{\partial x},$$

where ${D^{\prime }}_{0}$ represents the chloride diffusion coefficient in the pore solution of the cementitious materials and is about one order of magnitude smaller than $D_{0}$ [22]. In soil science, the influence of increased viscosity on diffusive flux is usually taken into account by a mobility factor. However, in the field of cementitious materials, this effect is always accounted for by using the pore solution diffusion coefficient ${D^{\prime }}_{0}$ in cementitious materials. In addition, the electrical double layer near the surface can hinder anion transport. This influence is significant when the pore size of the cementitious materials is comparable to the electrical double layer thickness [17]. This effect is also known as anion exclusion in soil science [23], and it can be accounted for by adding a factor γ in Equation (4). This effect is very difficult to separate from the tortuosity factor. It is appropriate to combine both effects and define a new factor, the apparent tortuosity factor $\tau_{a}$. Then, Equation (4) is rewritten as [20]

(5) $$J=-{D^{\prime }}_{0}\tau_{a}\theta \frac{\partial C}{\partial x},$$

where the apparent tortuosity factor $\tau_{a}$ is defined by $\tau_{a}=\tau \gamma$.

### 2.2. Effective Diffusion Coefficient

In the literature, the term effective diffusion coefficient is frequently used to describe the chloride transport property of cementitious materials [14,15]. It expresses the chloride transport property with the assumption that chloride ions do not interact with the solid reaction products. However, the effective diffusion coefficient is not a definite concept. It can be defined either by

(6) $$D_{e}={D^{\prime }}_{0}\tau_{a},$$

or by

(7) $${D^{\prime }}_{e}={D^{\prime }}_{0}\tau_{a}\theta ,$$

where $D_{e}$ and ${D^{\prime }}_{e}$ can be called effective diffusion coefficients [20]. The different definitions of the effective diffusion coefficient used in some of the studies are displayed in Table 1. 

It is worth noting that some researchers call both $D_{e}$ and ${D^{\prime }}_{e}$ effective diffusion coefficients but further clarify them with more description. For example, Martin-Perez et al. [24] referred to $D_{e}$ as the effective diffusion coefficient with the chloride concentration expressed in the unit volume of cementitious materials and referred to ${D^{\prime }}_{e}$ as the effective diffusion coefficient with the chloride concentration expressed in the unit volume of the pore solution. In the literature, ${D^{\prime }}_{e}$, as expressed by Equation (7), has occasionally been referred to as the intrinsic diffusion coefficient [25,26]. To differentiate between the diffusion coefficients defined in Equations (6) and (7), $D_{e}$ and ${D^{\prime }}_{e}$ in this paper are called the effective diffusion coefficient and the intrinsic diffusion coefficient, respectively. With the clear definition of the effective diffusion coefficient, the diffusive flux in Equation (5) is then modified as

(8) $$J=-D_{e}\theta \frac{\partial C}{\partial x}.$$

**Figure 2:**
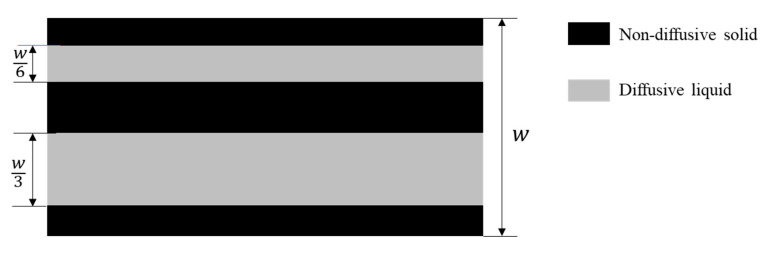
Schematic representation of a pore network system.

The difference between the effective and the intrinsic diffusion coefficient lies in whether or not to include the volumetric water content. The intrinsic diffusion coefficient is averaged over the entire cross-section of the cementitious material [26], while the effective diffusion coefficient is the diffusion coefficient in the pore system with the effect of pore system tortuosity. Figure 2 can be used to better understand the distinction between the effective diffusion coefficient and the intrinsic diffusion coefficient. In Figure 2, the porosity of the pore system is 0.5 and the tortuosity factor calculated from Equation (3) is 1. Therefore, the intrinsic diffusion coefficient ${D^{\prime }}_{e}$ is ${D^{\prime }}_{0}/2$, and the effective diffusion coefficient is still ${D^{\prime }}_{0}$.

**Table 1:** Definitions of effective diffusion coefficient in literature.

Definition of Effective Diffusion Coefficient	References
Equation (6)	[24,27,28]
Equation (7)	[14,15,24,28,29,30]

Because of the vague definition of the effective diffusion coefficient, special attention should be paid when interpreting this terminology in the literature. Otherwise, misinterpretation of the effective diffusion coefficient may cause an error of θ, which could easily exceed 50%.

### 2.3. Non-Steady Diffusion 

The chloride diffusion in cementitious materials is a time-dependent process. The mass conservation equation for this process can be obtained by considering the overall balance of chloride content over a representative element volume [31]. After some arrangement, this conservation equation is then written as:

(9) $$\frac{\partial \left(\theta C\right)}{\partial t}=-\frac{\partial J}{\partial x}+R,$$

where *J* is the diffusive flux calculated in Equation (8) and *R* (mol L^−1^ s^−1^) is the source term, expressed in the rate of supply or the removal of the chloride ions in the unit volume of cementitious material. When the volumetric water content θ is constant, θ can be removed from both sides of the equation. Equation (9) is then modified as follows, provided that the source term *R* is not considered

(10) $$\frac{\partial C}{\partial t}=D_{e}\frac{\partial^{2}C}{\partial x^{2}}.$$

It should be noted that Equation (10) has exactly the same form as Fick’s second law, but different meanings of “diffusion coefficient” apply in these two equations. In the original Fick’s second law, the diffusion coefficient represents the intrinsic property of the solute in the solution. While the “diffusion coefficient” in Equation (10) is the effective diffusion coefficient, and it incorporates several additional factors that influence the chloride diffusion, e.g., the tortuosity and increased viscosity.

When chloride diffuses in cementitious materials, the removal of chloride ions can occur due to physical adsorption and chemical reactions. Several reaction products in cementitious materials can bind a significant amount of chloride ions, such as C-S-H gel and monosulfate hydrate (AFm) in cement-based materials [32] and C-(N-)A-S-H gel and Mg-Al layered double hydroxide (LDH) phases in AAMs [33,34]. Hence, the chloride ions exist in two forms in cementitious materials. They are either bound by reaction products or free in the pore solution. It has been widely acknowledged that the initiation of rebar corrosion is only attributed to free (water-soluble) chloride ions [1]. As a result, chloride binding by reaction products will retard the corrosion process [35]. For chloride transport, the source term *R* in Equation (9) can be calculated using the chloride binding capacity:

(11) $$R=-\frac{\rho_{d}}{M_{Cl}}\frac{\partial C_{b}}{\partial t}$$

where $C_{b}$ (mg g^−1^) denotes the mass of bound chloride ions per mass of dry solid; $\rho_{d}$ (g cm^−3^) is the dry density of the cementitious materials; and $M_{Cl}$ (g mol^−1^) represents the molar mass of the chloride ions. With the assumption of homogenous materials and constant volumetric water content, the following equation can be obtained by substituting Equations (8) and (11) in Equation (9)

(12) $$\frac{\partial C}{\partial t}=D_{a}\frac{\partial^{2}C}{\partial x^{2}},$$

where *D_a_* represents the apparent diffusion coefficient and is expressed by

(13) $$D_{a}=\frac{D_{e}}{1+\frac{\rho_{d}}{\theta M_{Cl}}\frac{\partial C_{b}}{\partial C}}.$$

In Equation (13), the term $\partial C_{b}/\partial C.$ is introduced to account for the effect of chloride binding on the apparent diffusion coefficient. The determination of $\partial C_{b}/\partial C$ can be achieved from the chloride binding isotherm, which characterizes the relationship between free and bound chloride at different chloride concentrations [32]. In the literature, the non-linear binding isotherm is now widely accepted [32,36]. Therefore, $\partial C_{b}/\partial C$ varies with chloride concentration, and the apparent diffusion coefficient, as a result, is a changing variable dependent on the chloride concentration in the pore solution.

### 2.4. Definition and Unit of Related Variables

To correctly obtain the diffusion coefficient, extra attention should be paid to the unit and the definition of the involved variables in the process of deriving the diffusion coefficient. 

From Section 2.1, Section 2.2 and Section 2.3, the chloride concentration *C* is defined in the unit volume of the pore solution. This definition in the literature can be expressed in mol L^−1^ or kg m^−3^. In the above derivation, the chloride concentration *C* is expressed in mol L^−1^. When the chloride concentration *C* is represented in kg m^−3^, the apparent diffusion coefficient should be modified, and it can be calculated with Equation (14):

(14) $$D_{a}=\frac{D_{e}}{1+\frac{\rho_{d}}{\theta }\frac{\partial C_{b}}{\partial C}}.$$

In addition to defining the chloride concentration by its concentration in the pore solution, it is also often seen that the chloride concentration is defined in the unit volume of the cementitious materials, e.g., the chloride concentration in kg/m^3^ of the cementitious materials. To distinguish between these two definitions, the chloride concentration expressed in the volume of cementitious materials is denoted by $C^{*}$, and it is associated with concentration *C* by $C^{*}=\kappa \theta C$, where κ is a constant for unit conversion and depends on the unit of $C^{*}$ and *C*. When this definition applies, the diffusive flux in Equation (8) is rewritten as

(15) $$J=-D_{e}\frac{\partial C^{*}}{\partial x}.$$

Following the deriving process in Section 2.3, the governing equation for the non-steady diffusion process is then modified as

(16) $$\frac{\partial C^{*}}{\partial t}=D_{a}\frac{\partial C^{*2}}{\partial x},$$

where the apparent diffusion coefficient is expressed by

(17) $$D_{a}=\frac{D_{e}}{1+\frac{\rho_{d}}{\theta M_{Cl}}\frac{\partial C_{b}}{\partial C}}.$$

It should be pointed out that the chloride concentrations in Equations (16) and (17) are different. The concentration $C^{*}$ in Equation (16) is defined in the volume of cementitious materials, while concentration *C* in Equation (17) is expressed in the pore solution volume (mol L^−1^). This is because the chloride binding capacity depends on the pore solution concentration, not on the averaged chloride concentration in the cementitious materials.

During the derivation of the apparent diffusion coefficient, the unit of chloride binding capacity $C_{b}$ is sometimes also overlooked in the literature. $C_{b}$ is expressed in the literature as mg g^−1^, g g^−1^ and mol g^−1^, mmol g^−1^ of cementitious materials. Different units of $C_{b}$ will bring additional factors into the apparent diffusion coefficient. Some investigators may omit this factor. Such mistakes in the literature will be addressed in Section 4.

## 3. The Diffusion Coefficient Measured in Experiments

Many testing methods have been designed to obtain the chloride diffusion coefficient in cementitious materials. Based on different driving forces, these methods can be divided into two categories, namely natural diffusion tests and migration tests. In this study, we only focus on the diffusion coefficients determined by the natural diffusion tests. Two natural diffusion test methods are frequently employed to determine the diffusion coefficient in cementitious materials, i.e., the immersion test and the diffusion cell test. 

### 3.1. Immersion Test

The immersion test is a non-steady-state diffusion test. This method has been standardized in Nordtest NT Build 443 [5] and ASTM C1556 [6]. To ensure one-dimensional diffusion in this test method, only one surface of the test specimen was exposed to the chloride solution. All the other surfaces were sealed. The specimen was kept fully saturated before immersion to prevent capillary adsorption. The saturated specimen was then immersed in a concentrated chloride concentration (165 g NaCl per liter of solution in both NT Build 443 and ASTM C1556) for at least 35 days. After the immersion, the thin layers parallel to the exposed surface were ground off. The chloride profile inside the specimen was obtained by measuring the total chloride content in each layer. The following error function was then employed to fit the obtained chloride profile and thus the diffusion coefficient was determined,

(18) $$C^{\prime }(x,t)={C^{\prime }}_{s}-\left({C^{\prime }}_{s}-{C^{\prime }}_{i}\right)\cdot erf\left(\frac{x}{\sqrt{4\cdot D_{nssd}\cdot t}}\right),$$

where $C^{\prime }\left(x,t\right)$ (mass %) denotes the total chloride content at a specific depth *x* and after a certain exposure time *t*; ${C^{\prime }}_{s}$ (mass %) represents the chloride content on the exposed surface; ${C^{\prime }}_{i}$ (mass %) is the initial chloride content; and $D_{nssd}$ (m^2^ s^−1^) is the non-steady-state diffusion coefficient fitted in the test. 

To understand $D_{nssd}$, the origin of Equation (18) was analyzed. With the assumption of constant diffusion coefficient $D_{nssd}$, Equation (18) is the analytical solution of the following equation:

(19) $$\frac{\partial C^{\prime }}{\partial t}=D_{nssd}{\frac{\partial C^{\prime }}{\partial x}}^{2},$$

where the boundary condition is defined by $C^{\prime }(t=0)={C^{\prime }}_{i}$ (initial chloride concentration) and $C^{\prime }(x=0)={C^{\prime }}_{s}$ (constant surface concentration). It should be noted that Equation (19) is different from Equation (16) because $C^{\prime }$ in Equation (19) is the total chloride concentration while $C^{*}$ is the free chloride concentration. However, by analysis, it was found that Equation (19) could be transformed from Equation (16) under the assumption of the linear chloride binding isotherm. Appendix A shows a detailed derivation of Equation (19) from Equation (16). This sufficiently demonstrates that the diffusion coefficient measured in the immersion test represents the apparent diffusion coefficient $D_{a}$ under the assumption of the linear chloride binding isotherm.

### 3.2. Diffusion Cell Test

**Figure 3:**
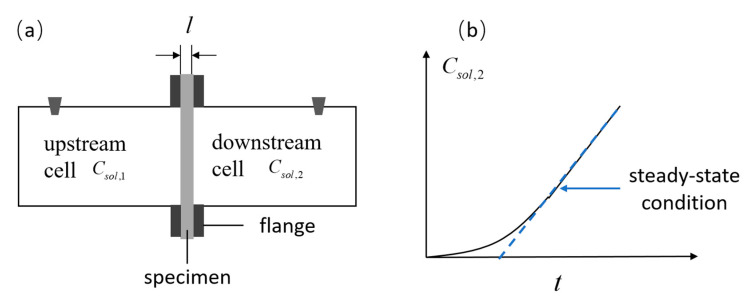
(**a**) Typical setup for diffusion cell test; (**b**) schematic illustration of time-dependent chloride concentration recorded in the downstream cell.

Unlike the immersion test, the diffusion cell test belongs to the steady-state diffusion test. In the literature, this test method is frequently employed to measure the diffusion coefficient [7,8]. A typical setup for the diffusion cell test is illustrated in Figure 3a. In the test, a cylindrical specimen separated two cells, i.e., the upstream cell and the downstream cell. The upstream cell was filled with concentrated chloride solution. In the downstream cell, the chloride concentration was chosen to be virtually zero. The upstream chloride concentration, $C_{sol,1}$, was kept constant by regularly replacing the solution with a new chloride solution. The downstream chloride concentration, $C_{sol,2}$, was continuously monitored. When the downstream chloride concentration shows a linear increase over time, it denotes that the steady state of chloride diffusion has been reached, and it also represents the end of the test (Figure 3b).

Using the monitored $C_{sol,2}$ and the other parameters in the diffusion cell test, the diffusive flux can be calculated by

(20) $$J_{cell}=\frac{V_{2}}{A}\frac{\Delta C_{sol,2}}{\Delta t},$$

where $V_{2}$ is the downstream cell volume, *A* is the specimen’s cross-section area, and $\Delta C_{sol,2}/\Delta t$ is the constant chloride concentration change rate in the downstream cell. Based on Fick’s first law, the diffusive flux in the diffusion cell test can be estimated by

(21) $$J_{cell}=-D_{cell}\frac{\partial C}{\partial x}=-D_{cell}\frac{C_{sol,2}-C_{sol,1}}{l},$$

where $D_{cell}$ is the diffusion coefficient measured in the diffusion cell test, and *l* represents the thickness of the cylindrical specimen. Then, $D_{cell}$ can be derived from Equations (20) and (21), as follows

(22) $$D_{cell}=\frac{V_{2}}{A}\frac{\Delta C_{sol,2}}{\Delta t}\frac{l}{C_{sol,1}-C_{sol,2}}$$

Due to the unclear definitions related to the diffusion coefficient in the literature, the measured diffusion coefficient $D_{cell}$ was examined carefully. Comparing Equation (21) to Equation (8), the chloride concentrations were both defined in the pore solution, and the diffusive flux also had the same definition. Therefore, the diffusion coefficient determined by the diffusion cell test is

(23) $$D_{cell}=D_{e}\theta$$

which is the intrinsic diffusion coefficient. This means that the diffusion coefficient measured by the diffusion cell test incorporated the influence of the apparent tortuosity, decreased the transport area, and increased the solution viscosity in the cementitious materials. 

It should be noted that $D_{cell}$ does not include the effect of the chloride binding. This is because it is assumed that the chloride binding is fully accomplished when the test reaches the steady-state condition. The numerical result also showed that the measured $D_{cell}$ was not influenced by the chloride binding. As long as the steady-state condition was reached, the measured value remained accurate even in the presence of the chloride binding [26]. 

The above discussion clearly shows the difference between $D_{cell}$ and $D_{nssd}$. They both depend on the tortuosity and the increased solution viscosity. However, $D_{cell}$ includes the influence of the decreased diffusive cross-section area and also excludes the chloride binding effect. Such differences should always be kept in mind when interpreting the diffusion coefficient determined from these two test methods.

## 4. Typical Errors 

Because of the vague definition of the diffusion coefficient and some misinterpretation of the experimentally determined diffusion coefficient, errors are frequently observed in the literature. These errors can be categorized according to three causes, as follows.

### 4.1. Neglecting Unit Conversion 

As explained in Section 2.3, the apparent diffusion coefficient, based on the effective diffusion coefficient, additionally takes into account the influence of the chloride binding. However, when calculating the apparent diffusion coefficient, such an influence is often miscalculated due to the frequent neglect of the unit conversion. In 2013, Zhang [16] numerically studied chloride transport in cement-based materials (CEM I 42.5 N). He calculated the apparent diffusion coefficient with

(24) $$D_{a}=\frac{D_{e}}{1+\frac{\partial C_{b}}{\partial C}}.$$

The influence of the chloride binding ($D_{a}/D_{e}$) calculated by Zhang is displayed in Figure 6.25 in [16]. In Equation (24), the units for these variables used by Zhang [16] are the same as the variables in Section 2, where *C* (mol L^−1^) is defined as the free chloride concentration in solution, and $C_{b}$ (mg g^−1^) is the chloride binding capacity. From the dimensional analysis, it is clearly shown that Equation (24) is wrong. $\partial C_{b}/\partial C$ should be dimensionless, while it is not in Equation (24) because $C_{b}$ and *C* have different units.

Comparing Equation (24) with Equation (13), it is noted that Zhang [16] has missed the term $\rho_{d}/\left(M_{Cl}\theta \right)$. For Portland cement paste, this term can range from 0.3 to 2, which causes a wrong calculation of the apparent diffusion coefficient.

A similar mistake also occurred in the study by Mundra et al. [37]. By employing the thermodynamic calculation of phase assemblage, Mundra et al. [37] developed an interactive software framework to estimate the chloride ingress into alkali-activated slag. In their study, the apparent diffusion coefficient is calculated as (Equation (7) in [37])

(25) $$D_{a}=\frac{D_{e}}{1+\frac{1}{\theta }\frac{\partial C_{b}}{\partial C_{f}}},$$

where the concentration of free chloride *C_f_* is expressed in kg/m^3^ of pore solution. Based on Equation (25), Mundra et al. show the influence of chloride binding on chloride ingress in Figure 6 in [37]. Equation (25) can be correct if $C_{b}$ is also defined as kg/m^3^ of cementitious materials. However, Mundra et al. [37] never explicitly described the definition of $C_{b}$ and always displayed $C_{b}$ as mg g^−1^ in the figures of the chloride binding isotherm. To confirm the units of $C_{b}$ and *C_f_* that Mundra et al. [37] used in Equation (25), the fitted Freundlich binding isotherm (Table 2 in [37]) was checked. It turned out that the actual units for $C_{b}$ and *C_f_* that Mundra et al. [37] used in Equation (25) were mg g^−1^ and mol L^−1^. In this case, the correct equation to calculate the apparent diffusion coefficient for Mundra et al. [37] should be

(26) $$D_{a}=\frac{D_{e}}{1+\frac{\rho_{d}}{\theta M_{Cl}}\frac{\partial C_{b}}{\partial C}}.$$

Comparing Equation (25) with Equation (26), it was found that Mundra et al. [37] had missed the term $\rho_{d}/M_{Cl}$, which was around 0.06. This mistake significantly overestimated the impact of the chloride binding on chloride transport. Meanwhile, it also caused an underestimation of the apparent diffusion coefficient.

### 4.2. Misunderstanding of Effective Diffusion Coefficient

Because of the vague definition of the effective diffusion coefficient in the literature, errors can result from the misunderstanding of this concept.

It is the effective diffusion coefficient (Equation (6)) that should be used in the calculation of the apparent diffusion coefficient (Equation (13)). However, some researchers misused the intrinsic diffusion coefficient (Equation (7)). Zhang [16] intended to numerically calculate the apparent diffusion coefficient for Portland cement paste. When he calculated $D_{a}$, the effective diffusion coefficient was derived from:

(27) $$D_{e}^{zhang}=\frac{J}{J_{0}}{D^{\prime }}_{0},$$

where *J* represents the diffusive flux across the cementitious material’s cross-section, and *J*_0_ is the diffusive flux in the free pore solution under the same boundary condition. The calculated effective diffusion coefficient by Zhang is displayed in Figure 6.19 and Figure 6.21 in [16]. By applying Equation (5) and $J_{0}={D^{\prime }}_{0}\partial C/\partial x$ to Equation (27), it can be found that the “effective” diffusion coefficient calculated by Zhang [16] is actually the intrinsic diffusion coefficient. This misunderstanding of the effective diffusion coefficient by Zhang [16] leads to an underestimation of the apparent diffusion coefficient by θ, i.e., $D_{a}^{zhang}/D_{a}=\theta$, where $D_{a}^{zhang}$ is the apparent diffusion coefficient calculated in [16]. 

Such misunderstanding can also result in an incorrect governing equation. A numerical investigation was carried out by Jiang et al. [38] on chloride diffusion in concrete affected by freeze–thaw cycles. In their study, the following equation (Equation (9) in [38]) is used as a governing equation to numerically obtain the chloride content inside the concrete

(28) $$\frac{\partial C}{\partial t}=D_{k}\nabla^{2}C,$$

where *C* is defined as chloride concentration in the pore solution. *D_k_* in [38] represents the diffusion coefficient of the *k*th phase, where *k* = 1, 2, 3 denotes cement paste, interfacial transition zone, and aggregate, respectively. Clearly, the term “diffusion coefficient” is a vague expression to readers. It can refer to the effective diffusion coefficient or the intrinsic diffusion coefficient defined in this article. According to Equations (6) and (7) in [38], *D_k_* is inferred as the intrinsic diffusion coefficient. However, it can be seen from Equation (10) that *D_k_* in Equation (28) should be the effective diffusion coefficient if Equation (28) is a correct governing equation. The misunderstanding of the diffusion coefficient results in the use of the intrinsic diffusion coefficient in the place where the effective diffusion coefficient should be used. Because of this mistake, the results of the chloride concentration obtained from the numerical simulation in [38] have all been underestimated. 

### 4.3. Comparing with the Wrong Experimental Result

The experimental results from the immersion test method or diffusion cell test method are usually used to validate modeled diffusion coefficients. As discussed in Section 3, the diffusion coefficients measured from these two methods are different. To effectively validate the numerical results, an appropriate test method should be chosen. However, insufficient understanding of the non-steady-state immersion test and the steady-state diffusion cell test can lead to the ineffective validation of numerical results. Gu et al. [39] employed the finite element method to model the chloride diffusion coefficient in ultra-high performance concrete (UHPC) paste which is composed of Portland cement, fly ash, and silica fume. The diffusion coefficient of UHPC paste in their model is calculated by

(29) $$D_{e}^{gu}=\frac{Q}{A}\frac{L}{\Delta C},$$

where *Q* represents the chloride diffusion mass rate; *A* is the cross-section area of UHPC paste; *L* represents the simulated sample length; and ΔC is the chloride concentration difference between both ends. Table 5 in [39] shows the comparison of the chloride diffusion coefficient calculated by numerical simulation with that measured by experiment.

According to the previous analysis in Section 2, the diffusion coefficient $D_{e}^{gu}$ that Gu et al. [39] calculated is the intrinsic diffusion coefficient. To validate their results, the immersion test based on Nordtest NT Build 443 was carried out, and the apparent diffusion coefficient was obtained. Gu et al. [39] then validated their modeled intrinsic diffusion coefficient with the apparent diffusion coefficient measured from the experiment, which was obviously a false validation. The theoretical ratio between these two diffusion coefficients is:

(30) $$\frac{D_{e}^{gu}}{D_{a}}=\theta \left(1+\alpha \frac{\partial C_{b}}{\partial C}\right),$$

where α is a unit conversion factor based on the units used for $C_{b}$ and *C*. When a clear understanding is established for the experimentally measured diffusion coefficient, it should be found that the steady-state diffusion cell test is the one that exactly fits their validation purposes. Therefore, an effective validation of the modeled results in [39] can be accomplished by the diffusion cell test.

## 5. Conclusions

In this paper, the equations that describe chloride diffusion in cementitious materials are presented. The influencing factors on the chloride diffusion are identified and include tortuous pathways, reduced diffusive cross-section area, increased viscosity of pore solution, and anion exclusion. This paper highlights that the term “effective” diffusion coefficient, which is commonly found in the literature, can be defined in two distinct ways. This paper uses the terms “effective diffusion coefficient” and “intrinsic diffusion coefficient” to refer to these two definitions. The factors considered in the effective diffusion coefficient are the tortuous pathways and the increased solution viscosity. In contrast, the intrinsic diffusion coefficient additionally incorporates the effect of the reduced diffusive cross-section area. The chloride ions in the pore solution can be bound by the reaction products of the cementitious materials. To include the influence of chloride binding on the diffusion coefficient, the apparent diffusion coefficient is introduced and clarified. The unit and definition of some variables, such as chloride concentration and chloride binding capacity, vary in the literature. It was shown that such variations can result in an unnoticeable change in the diffusion equation and in the calculation of the apparent diffusion coefficient. 

The diffusion coefficient measured in the two different natural diffusion test methods was then analyzed. The non-steady-state diffusion coefficient determined in the immersion test was demonstrated to be the apparent diffusion coefficient under the assumption of linear chloride binding. The steady-state diffusion coefficient determined in the diffusion cell test was the intrinsic diffusion coefficient, in which the chloride binding effect was not included. In the end, three typical errors related to the diffusion coefficient, which have occurred in the literature, were analyzed. These errors resulted from the neglect of the unit conversion, the vague definition of the effective diffusion coefficient, and the misunderstanding of the experimentally measured diffusion coefficient.

In summary, the unclear definitions and varied units related to chloride diffusion make the diffusion coefficient quite misleading. Caution should be exercised when dealing with the diffusion coefficient. Otherwise, misinterpretation and wrong calculation of the diffusion coefficient can easily happen.

## Data Availability

No new data were created or analyzed in this study. Data sharing is not applicable to this article.

## Appendix A. Derivation of Governing Equation in the Chloride Immersion Test

In the immersion test, e.g., NT Build 443 and ASTM C1556, the chloride diffusion coefficient was obtained by fitting the measured total chloride concentration $C^{\prime }$ with the analytical solution of Equation (19). To understand the measured diffusion coefficient in such a test, Equation (19) was derived from Equation (16).

The concentration of free chloride $C^{*}$ (kg/m^3^ of cementitious material) can be calculated with the total chloride concentration $C_{t}^{*}$ (kg/m^3^ of cementitious material) and binding content $C_{b}$ (mg g^−1^) by

(A1) $$C^{*}=C_{t}^{*}-\rho_{d}C_{b}.$$

Substituting Equation (A1) into Equation (16) gives:

(A2) $$\frac{\partial C_{t}^{*}}{\partial t}=D_{a}\frac{\partial^{2}C_{t}^{*}}{\partial x^{2}}+\rho_{d}\left(\frac{\partial C_{b}}{\partial t}-D_{a}\frac{\partial^{2}C_{b}}{\partial x^{2}}\right).$$

The chloride binding content is the function of free chloride concentration *C* in the pore solution; so, the right-most term in Equation (A2) can be rewritten as follows if the linear chloride binding isotherm applies:

(A3) $$\frac{\partial C_{b}}{\partial t}-D_{a}\frac{\partial^{2}C_{b}}{\partial x^{2}}=\frac{\partial C_{b}}{\partial C}\left(\frac{\partial C}{\partial t}-D_{a}\frac{\partial^{2}C}{\partial x^{2}}\right).$$

According to Equation (12), Equation (A3) is equal to 0, which turns Equation (A2) into

(A4) $$\frac{\partial C_{t}^{*}}{\partial t}=D_{a}\frac{\partial^{2}C_{t}^{*}}{\partial x^{2}}.$$

The chloride concentration $C^{\prime }$ in the immersion test is expressed in mass %. As a result, it gives $C_{t}^{*}=10\cdot \rho_{d}C^{\prime }$. Substituting this equation into Equation (A4) will generate the governing equation of the immersion test, Equation (19). This demonstrates that the diffusion coefficient $D_{nssd}$ measured in the immersion test represents the apparent diffusion coefficient $D_{a}$ with the assumption of the linear chloride binding isotherm.
